# The facilitation effect and language thresholds

**DOI:** 10.3389/fpsyg.2014.01197

**Published:** 2014-10-31

**Authors:** Kellie Rolstad, Jeff MacSwan

**Affiliations:** Teaching and Learning, Policy and Leadership, University of MarylandCollege Park, MD, USA

**Keywords:** bilingualism, biliteracy, Threshold Hypothesis, facilitation theory, language minority education

Lechner and Siemund (2014) set out to determine whether bilinguals have an advantage for learning additional languages over monolinguals, purporting to evaluate the Threshold Hypothesis of Cummins (1979a) in this context. The study investigated the attainment of English literacy by Turkish-German, Vietnamese-German, and Russian-German simultaneous and sequential bilinguals for whom English is a third language, and found significant correlations (at 0.01 level) for the second language (German) with the third language (English) at 0.580, and for the heritage language with the second language at 0.638; while the heritage language correlated with the third language at 0.277, the result was non-significant (*N* = 34). This crosslinguistic transfer effect is well-documented in the scholarly literature for first language (L1) and second language (L2) learners (Genesee et al., 2006; Goldenberg, 2011), but very little prior work has been done to examine crosslinguistic transfer of literacy among trilinguals. The Threshold Hypothesis specifically points to ability levels in the first language as the mechanism which facilitates attainment in the second language (extended to a third language for Lechner and Siemund). The primary conceptual problem with “ability” in the first language is that it lacks any grounded theoretical description of “levels,” and simply equates social status with linguistic ability much as classical prescriptivist ideology does (MacSwan and Rolstad, 2003, 2010; Wiley and Rolstad, 2014).

Cummins (1976) developed the Threshold Hypothesis to account for an apparent conflict in findings regarding the cognitive benefits of bilingualism. Earlier studies concluded that cognitive progress and school achievement were negatively affected by bilingualism, while more recent research appeared to show “positive cognitive consequences.” Cummins noted that the studies that found a negative effect involved linguistic minorities, and those finding a positive effect involved a condition of “additive bilingualism” in which linguistic *majority* children are learning an additional language. Cummins theorized that the linguistic minorities were undergoing loss of their first language, and that “the level of linguistic competence attained by a bilingual child may mediate the effects of his bilingual learning experiences on cognitive growth.” That is, reports of negative effects of bilingualism for “cognitive and scholastic progress” related to minority children's (hypothesized) lower level of linguistic proficiency in the first language, as affected by acquiring a second, while children in the “additive” bilingual programs had the benefit of continued support of their first language. As Cummins (1976) put it,

Subtractive bilingualism, where L1 [first language] is being replaced by L2 [second language], implies that as a bilingual in a language minority group develops skills in L2, his competence in L1 will decrease. It seems likely that, under these circumstances, many bilingual children in subtractive bilingual learning situations may not develop native-like competence in either of their two languages (p. 20).

In later work, Cummins (1979a) extended his analysis to another similar problem. Swain (1978) had made the case that immersion programs, in which linguistic majorities are (partially or totally) immersed in an L2, differ in important respects from submersion programs, in which language minority children are immersed in a majority language (Cohen and Swain, 1976; Swain, 1978). Today, considerable research on program effectiveness has borne out this expectation, as it shows that children in bilingual programs generally outperform similar children in English immersion programs in the US, and that children with more access to home language support do even better than children with less access (see Rolstad et al., 2005, and works cited there).

To address these observed differences, Cummins (1979b, p. 223) proposed “a theoretical framework which assigns a central role to the *interaction* between socio-cultural, linguistic and school program factors,” in which “the level of competence bilingual children achieve in their two languages acts as an intervening variable in mediating the effects of their bilingual learning experiences” (Cummins, 1976, p. 229). Background characteristics, child input factors, and educational treatment variables acted together to influence “child process variables,” in Cummins' theory, resulting in minority children's differential ability in L1 and L2. A potentially resulting condition of semilingualism is thus posited to explain academic achievement differences among children. Embedded in the Threshold Hypothesis,

negative cognitive and academic effects are hypothesized to result from low levels of competence in both languages or what Scandinavian researchers (e.g., Hansegård, 1968; Skutnabb-Kangas and Toukomaa, 1976) have termed “semilingualism” or “double semilingualism” … Essentially, the lower threshold level of bilingual competence proposes that bilingual children's competence in a language may be sufficiently weak as to impair the quality of their interaction with the educational environment through that language (1979a, p. 230).

In Cummins' theory, semilingualism is a potential characteristic of minority language children, but not of majority language children, and is the cause of their weaker academic performance. For children in an additive situation, semilingualism does not degrade the quality of interactions in the classroom, generally leading to school success.

Lechner and Siemund take care to note, based on critical discussion in MacSwan (2000a) and MacSwan and Rolstad (2006, 2010), that literacy and language are different constructs, and seek to remove the blemish of semilingualism from the Threshold Hypothesis. In doing so, the authors observe, “We do not regard the Threshold Hypothesis as a competence-related construct, but rather as a performance-based concept relating to educational attainment.” Elaborating,

We believe that it is necessary to distinguish between linguistic competence and performance (langue versus parole) in the interpretation of the Threshold Hypothesis. Cummins seems to use the term “language proficiency” to refer to both competence and performance, including school literacy. MacSwan (2000a, pp. 33–34) argues that if the Threshold Hypothesis refers to language competence, it is spurious because there is no evidence to suggest that subtractive bilinguals did not know the underlying principles of their language.

However, the competence/performance distinction does not help in a general way to overcome the conceptual weaknesses of the Threshold Hypothesis. Competence refers to linguistic knowledge, and performance to the use of that knowledge in concrete, everyday situations (Chomsky, 1965). We utter things all the time which we immediately recognize to be ill-formed, reflecting on our linguistic competence, due to fatigue, distraction, memory loss, or other language-external factors. Because our underlying system of competence relies on recursive generative rules, it can theoretically produce sentences that are infinitely long; but as finite beings, we can't stick around long enough to say them. Claiming that “ability” levels differ according to linguistic performance rather than linguistic competence seems to achieve little or nothing, and still demands supporting evidence, just as it did when these differences were conceptualized in terms of linguistic competence. And as before, in Cummins' original proposals, relevant evidence is lacking, and other work indicates that the hypothesized “levels” are not to be found (MacSwan et al., 2002; MacSwan and Rolstad, 2006).

Granted, “linguistic performance” is used as a large, undifferentiated container of many very different kinds of psychological phenomena which competence-focused linguists generally wish to set aside, and context-sensitive language use, such as pragmatics and discourse, might reasonably be regarded as governed by the theory of linguistic performance, in part. Indeed, Cummins provides a four-component definition of “language proficiency,” following Canale and Swain (1980) and Canale (1983): Grammatical competence, sociolinguistic competence, discourse competence, and strategic competence. Cummins' (1981) definition of “sociolinguistic competence” is similar to what Chomsky (1978, p. 224) called “pragmatic competence,” defined as “knowledge of conditions and manners of appropriate use, in conformity with various purposes.” For Cummins (1981), discourse competence consists of “knowledge of how to combine meanings and forms to achieve a unified text in different modes” (p. 7), and strategic competence is the “mastery of verbal and non-verbal strategies” which assist under conditions of breakdowns in other competence domains. In this broader context, Cummins (1981) settled on a framework in which “literacy is viewed as one aspect of communicative proficiency” (p. 14).

If we think of school-based literacy and the particular language used at school as a different language register, or a Discourse in Gee's (1996) sense, then we are well-positioned to characterize school language as domain- and place-focused—the language of school differs from the language of skateboarding just as the language of boatbuilding differs from the language of farming. But notice that this is not the concept of language proficiency embedded in the Threshold Hypothesis, where groups are said to differ by ability levels, not contexts, and so the appeal to linguistic performance, or even context of language use, does not help to avoid the prescriptivist character inherent in the model.

Rather than try to salvage the Threshold Hypothesis, we suggest that an alternative theoretical framework be pursued, and that the original Threshold Hypothesis be discarded. In our own work, we have advocated the facilitation theory (MacSwan and Rolstad, 2005)—the view that cognitive architecture permits and facilitates transfer of literacy cross-linguistically precisely because it is essentially language-external. This view is also consistent with the approach in Riches and Genesee (2006), who posit “a common underlying reservoir of literacy abilities” available to L2 learners who are good L1 readers.

As argued in MacSwan and Rolstad (2005), the neuropsychological evidence suggests that language is separate and discrete from other mental faculties, taking psychological modularity as our framework (as is typical in the cognitive neurosciences). In the case of bilingualism, both languages are represented in the human language faculty (Epstein et al., 1996; MacSwan, 2000b, 2014). But unlike language and other perceptual systems, western-style literacy is a recent invention, and is absent from many human cultures (MacSwan, 2000a; Gee, 2001; MacSwan and Rolstad, 2010).

In fact, literacy seems to rely on a wide range of cognitive faculties; besides knowledge of language, these include background knowledge, systems of reasoning, motor control, visual processing, shape recognition, and context. Reading and writing are independent of special-purpose mental faculties like language, and should be conceptualized as a technological invention. Thus, literacy recruits knowledge as needed from relevant cognitive systems. Evidence from cases of selective impairment in which a blow to the posterior regions of the brain renders a person suddenly unable to read but with all normal language faculties intact adds additional support to this view (Gardner, 1974). School literacy may therefore be seen as one of several ways language is used to satisfy human purposes; it uses linguistic and other cognitive resources to represent language, but it is not itself an aspect of language ability in the linguistic sense.

We suggest, then, that “transfer” of literacy across languages occurs because in the bilingual brain, both languages have access to the same cognitive resources. As Genesee et al. (2006) observe in a comprehensive review, transfer of first language literacy to the second language context is found in studies of word reading (across age, linguistic typology, and L2 proficiency), reading comprehension (across age, typology, language status, direction of transfer, and tasks), and reading strategies. They found phonological processes underlying word recognition to be influenced by orthography, but a facilitation effect was still present. (We note that two of the three heritage language groups assessed in Lechner and Siemund's study—Vietnamese and Russian—use non-Latinate orthographies, contrasting with English and German, and the sample size of the Turkish-background group was relatively very small, at *N* = 5; these factors may underlie the positive but non-significant result for the correlation of heritage language literacy with English literacy in Lechner and Siemund's results.)

It is apparent, then, that one does not see a relationship between “first language ability,” however that is to be conceived, and second language literacy, but between literacy in the first language and literacy in the second language; in other words, literacy is relatively independent of the particular language for which it was initially acquired. In this regard, the usual meaning of transfer, which implies that a process “moves” knowledge from one language to another, should be seen as strictly a metaphor: Rather than move, both languages have access to the same store of knowledge, available to learners regardless of how the knowledge was acquired in the first place, as Riches and Genesee (2006) also suggested. Access to knowledge acquired in a first language has a facilitation effect on learning in the second language context.

## Conflict of interest statement

The authors declare that the research was conducted in the absence of any commercial or financial relationships that could be construed as a potential conflict of interest.
